# Cervical cord myelin water imaging shows degenerative changes over one year in multiple sclerosis but not neuromyelitis optica spectrum disorder

**DOI:** 10.1016/j.nicl.2017.06.019

**Published:** 2017-06-16

**Authors:** Anna J.E. Combes, Lucy Matthews, Jimmy S. Lee, David K.B. Li, Robert Carruthers, Anthony L. Traboulsee, Gareth J. Barker, Jacqueline Palace, Shannon Kolind

**Affiliations:** a Department of Neuroimaging, Institute of Psychiatry, Psychology & Neuroscience, King's College London, London, UK; b Nuffield Department of Clinical Neurosciences, University of Oxford, Oxford, UK; c Department of Radiology, Faculty of Medicine, University of British Columbia, Vancouver, Canada; d Division of Neurology, Faculty of Medicine, University of British Columbia, Vancouver, Canada

**Keywords:** Multiple sclerosis, Neuromyelitis optica, Spinal cord, Magnetic resonance imaging, Myelin water imaging, Longitudinal study

## Abstract

Spinal cord pathology is a feature of both neuromyelitis optica spectrum disorder (NMOSD) and relapsing-remitting multiple sclerosis (MS). While subclinical disease activity has been described in MS using quantitative magnetic resonance imaging measures, current evidence suggests that neurodegeneration is absent between relapses in NMOSD, although most evidence comes from brain studies. We aimed to assess cross-sectional differences and longitudinal changes in myelin integrity in relapse-free MS and NMOSD subjects over one year. 15 NMOSD, 15 MS subjects, and 17 healthy controls were scanned at 3 T using a cervical cord mcDESPOT protocol. A subset of 8 NMOSD, 11 MS subjects and 14 controls completed follow-up. Measures of the myelin water fraction (f_M_) within lesioned and non-lesioned cord segments were collected. At baseline, f_M_ in lesioned and non-lesioned segments was significantly reduced in MS (lesioned: p = 0.002; non-lesioned: p = 0.03) and NMOSD (lesioned: p = 0.0007; non-lesioned: p = 0.002) compared to controls. Longitudinally, f_M_ decreased within non-lesioned cord segments in the MS group (− 7.3%, p = 0.02), but not in NMOSD (+ 5.8%, p = 0.1), while change in lesioned segments f_M_ did not differ from controls' in either patient group. These results suggest that degenerative changes outside of lesioned areas can be observed over a short time frame in MS, but not NMOSD, and support the use of longitudinal myelin water imaging for the assessment of pathological changes in the cervical cord in demyelinating diseases.

## Highlights

• MS and NMOSD subjects underwent longitudinal cervical cord myelin water imaging.
• Reduced myelin water fraction in MS and NMOSD normal-appearing and lesioned areas
• Decrease in myelin in normal-appearing tissue over 1 year in MS, but not NMOSD
• Further evidence that disease progression is absent between relapses in NMOSD.

## 1 Introduction

Neuromyelitis optica spectrum disorder (NMOSD) is a relapsing autoimmune disease of the central nervous system that, due to similar clinical and neurological features, was long thought to be a rare variant of multiple sclerosis (MS) (Wingerchuk et al., 2015). Since the discovery of a highly specific antibody (Jarius and Wildemann, 2010), and the advent of serum testing to aid differential diagnosis, it is now considered a separate entity (Weinshenker, 2007). Serum antibodies to the aquaporin-4 water channel protein (AQP4-Ab), found on astrocytic foot processes, are detectable in a high proportion of patients (Pandit et al., 2015). AQP4 is expressed throughout the brain, and is found in particularly high concentration in the optic nerve and spinal cord, in line with the observed frequency of pathology in these regions in NMOSD (Pittock et al., 2006).

Unlike in MS, which can present as a relapsing-remitting disease with secondary conversion to a progressive phase, or as progressive from onset (Compston and Coles, 2008), conversion to a progressive phase is extremely rare in NMOSD (Aboul-Enein et al., 2013, Cabre et al., 2009, Collongues et al., 2010, Collongues et al., 2014). Clinical disability is accrued as a consequence of damage sustained during relapses, whereas clinical disability scores in MS increase more steadily during the progressive phase (Collongues et al., 2011, Wingerchuk et al., 2007a). Current clinical and neuroimaging evidence suggests that subclinical disease activity does not occur between attacks in NMOSD (Wingerchuk et al., 2007b), contrary to what is observed in MS (Filippi and Agosta, 2010, Matthews et al., 2015). However, it has been suggested that NMOSD attacks are so severe that the resulting sequelae hide the subtler changes that may accrue over time as a result of progressive axonal deterioration following inflammation (Wingerchuk et al., 1999).

The cervical spinal cord is a frequent target of disease activity in both NMOSD and MS. The main feature of cord pathology in NMOSD is the presence of longitudinally extensive lesions, spanning three or more vertebral segments. These favour the grey matter (Krampla et al., 2009, Nakamura et al., 2008) and are characterised by abnormal magnetic resonance imaging (MRI) diffusion metrics, reflecting greater tissue injury compared with MS lesions (Klawiter et al., 2012, Rivero et al., 2014). Abnormal magnetization transfer (Benedetti et al., 2006, Filippi et al., 1999, Rocca et al., 2004) and diffusion parameters (Jeantroux et al., 2012, Pessôa et al., 2012, Qian et al., 2011, Rivero et al., 2014) have been observed in the cervical cord in NMOSD, suggesting the presence of inflammatory processes, demyelination and axonal pathology. Abnormally low myo-inositol (normalized to creatine) levels have been measured in the upper cervical cord, thought to reflect astrocytic dysfunction within lesions, a process thought to play a major role in the pathogenesis of the disease by contributing to oligodendrocyte dysfunction and eventually secondary demyelination (Ciccarelli et al., 2013).

Overall, focal cord pathology is considered more aggressive in NMOSD; diffuse damage of the type seen in MS has only been shown inconsistently, while secondary degenerative processes in white matter tracts may be common to both diseases (Klawiter et al., 2012). However, the majority of studies do not differentiate between lesional and normal-appearing spinal cord tissue (NASCT); to date, no longitudinal advanced imaging study has assessed whether changes in NASCT occur in NMOSD outside of clinical relapses.

Multicomponent Driven Equilibrium Single Pulse Observation of T_1_ and T_2_ (mcDESPOT) is a quantitative myelin water imaging method with great sensitivity for the estimation of myelin content (Deoni et al., 2008), and has been suggested as a possible marker of disease progression in primary progressive MS (Kolind et al., 2015). Matthews et al. (2015) previously found no evidence of disease progression in a group of clinically stable NMOSD subjects, while several quantitative imaging brain metrics, including the mcDESPOT-derived myelin water fraction (f_M_) in major white matter tracts, showed widespread differences and changes over one year in a group of relapsing-remitting MS subjects. In the present study, we report cross-sectional and longitudinal evaluations of the cervical spinal cord in a subset of the same NMOSD, MS subjects and healthy controls using mcDESPOT. We aimed to characterise normal-appearing and lesional cervical cord pathology at baseline, and to assess whether evidence of degenerative changes could be detected over one year in either patient group.

## 2 Methods

### 2.1 Population characteristics and study design

#### 2.1.1 Ethics

This study was approved by the South East Hampshire NHS Research Ethics Committee. All participants gave written informed consent before taking part.

#### 2.1.2 Subjects

15 AQP4-Ab NMOSD patients, 15 relapsing-remitting MS patients, and 17 sex and age-matched healthy controls were recruited from the NHS Neuromyelitis Optica Highly Specialized Service in Oxford, UK – a subset of the groups previously reported on in Matthews et al. (2015). All NMOSD subjects were receiving immunosuppressant medication (7 on azathioprine, 2 on methotrexate, 1 on prednisone, and 5 on combinations thereof), while the majority of MS subjects were on disease-modifying therapies (6 on Copaxone, 3 on beta-interferons, and 1 on low-dose naltrexone; 5 were not receiving treatment). A subset of 8 NMOSD, 11 MS and 14 controls completed a follow-up scan after one year. All patients had been relapse-free for at least 6 months prior to the baseline scan, and none experienced a relapse between baseline and follow-up.

#### 2.1.3 MRI acquisition

Participants were scanned on a 3 Tesla MRI scanner (Siemens MAGNETOM Verio, Erlangen, Germany) with a mcDESPOT protocol (Kolind and Deoni, 2011) which covered the whole cervical cord with 0.9 × 0.9 × 1.8 mm voxels, reconstructed to 0.9 mm^3^ isotropic (scan time 22 min). The mcDESPOT data consisted of series of spoiled gradient echo (SPGR) scans over a range of 8 optimized flip angles (α) (TE/TR = 2.7/6.1 milliseconds (ms); α = [2.25, 4.5, 6.75, 9, 11.25, 13.5, 15.75, 18]°), 8 balanced steady state free precession scans (TE/TR = 2.3/4.6 ms; α = [5.625, 11.25, 16.875, 22.5, 28.125, 33.75, 39.375, 45]°) acquired over two phase-cycling patterns (0° and 180°) to correct for off-resonance effects (Deoni, 2009), and an inversion recovery-prepared SPGR scan for correction of B_1_ inhomogeneity (Deoni, 2011) (TE/TR = 2.7/6.3 ms, TI = 450 ms, α = 5°). An axial T_2_-weighted multi-echo gradient echo sequence, sagittal T_1_-weighted magnetization-prepared rapid gradient echo, and a sagittal T_2_-weighted turbo spin echo sequence were acquired for lesion assessment.

### 2.2 Image analysis

#### 2.2.1 Lesion identification

Lesions were identified on the patients' anatomical scans by an experienced radiologist (J.S.L.) blinded to diagnosis, primarily based on the sagittal T_2_-weighted scan with additional information gleaned from the sagittal T_1_ and axial T_2_-weighted scans. The assessment was done for both baseline and follow-up concurrently. Spinal levels (heretofore referred to as segments) were identified as normal-appearing or lesioned. For small lesions located at a disc level, both adjoining segments were considered lesioned.

#### 2.2.2 Segmentation

**Fig. 1:**
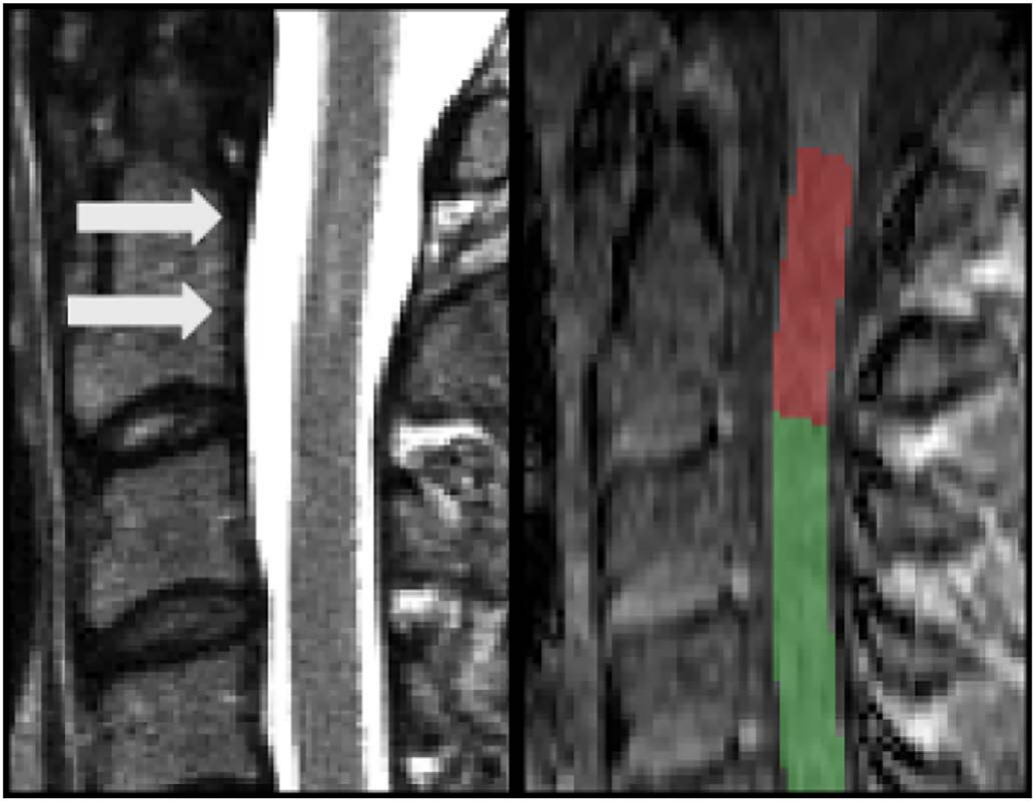
Example of a T_2_-hyperintense lesion (arrows) in an NMOSD patient at the C1/C2 level (left), and cord segmentation on an SPGR image from the mcDESPOT protocol (right). The area at the C1/C2 level was categorized as lesioned (in red), and the remaining portion of the cord as preserved tissue (in green). (For interpretation of the references to color in this figure legend, the reader is referred to the web version of this article.)

For each subject, an SPGR image from the mcDESPOT protocol with good contrast between tissue and cerebrospinal fluid (α = 9°) was used for preprocessing. The spinal cord was segmented using *PropSeg* (De Leener et al., 2014), a semi-automated propagation-based method from the Spinal Cord Toolbox (De Leener et al., 2016) (SCT; http://sourceforge.net/projects/spinalcordtoolbox/). Each subject's SPGR was warped to the MNI-Poly-AMU template (Fonov et al., 2014). The inverse transform was then applied to the template in order to obtain vertebral level segmentation in subject space. We considered the region from C1 to C7 for whole cervical cord measures. Using the lesion assessment described above, separate masks were created by considering only segments marked as either normal-appearing or lesioned. An example of a lesioned tissue mask is shown in Fig. 1.

#### 2.2.3 f_M_ measurement

Images from the mcDESPOT protocol were linearly registered within-subject to the reference SPGR scan with FSL-FLIRT (Jenkinson et al., 2002), using trilinear interpolation. f_M_ maps were calculated with a three-pool model (Deoni et al., 2013), and manually edited (by an observer blinded to group and time point (A.J.E.C.)) to exclude voxels where partial volume effect in the acquired images led to artificially very low computed values. Visual inspection was performed for all images to ensure the quality of co-registration. Median f_M_ values were collected within the whole cervical cord, and within NASCT and lesioned tissue separately using the masks described above.

### 2.3 Statistics

Non-parametric tests were chosen due to small sample sizes, and after visual inspection showed that MRI variables were not normally distributed. Percent changes between baseline and follow-up metrics were calculated for each subject. Differences between patient groups were evaluated using the Mann-Whitney *U* test, and between the three groups using the Kruskal-Wallis test. Post hoc comparisons following a significant omnibus test (alpha = 0.05) were conducted with the Mann-Whitney *U* test. Within-group differences were assessed using paired Wilcoxon's signed rank test; we tested cross-sectional differences between NASCT and lesional segments within each patient group, and changes over time within each group and tissue type. Results were not corrected for multiple comparisons, due to the exploratory nature of this study. Statistical analysis was conducted in R version 3.3.3 (R Core Team, 2017).

## 3 Results

Subject characteristics are summarized in Table 1. There was no significant difference in age between the three groups (p = 0.6). Both patient groups had equivalent disease duration (p = 0.6). The NMOSD group had a higher median EDSS (p = 0.03). Summary baseline and longitudinal MRI metrics, and group comparisons are displayed in Table 2.

**Table 1:** Population characteristics for the cross-sectional sample (median (range)). Group comparisons are performed with the Kruskal-Wallis test. Where only pairwise MS vs. NMOSD comparisons are appropriate, results from the Mann-Whitney *U* test are reported.

	Controls	MS	NMOSD	p
N (Sex)	17 (4 M)	15 (4 M)	15 (3 M)	–
Age (years)	54 (19–76)	41 (22–68)	45 (20–76)	0.6
Disease duration (months)	–	72 (24–254)	60 (12–186)	0.6
Baseline EDSS	–	2 (0–5)	4 (2–7.5)	0.03⁎
Lesioned segments per subject	–	5 (0–7)	3 (0–7)	0.1
Number of subjects with NASCT	–	11	13	–
Number of subjects with ≥ 1 lesion	–	14	12	–
Number of subjects with no lesions	–	1	3	–

NASCT: normal-appearing spinal cord tissue.

⁎ Significant at p ≤ 0.05.

**Table 2:** Median (interquartile range) baseline values and percent change in MRI metrics.

	Controls	MS	NMOSD	Kruskal-Wallis	MS vs. controls	NMOSD vs. controls	NMOSD vs. MS
Cross-sectional							
NASCT f_M_	0.159 (0.017)	0.148 (0.016)	0.149 (0.015)	0.006⁎⁎	0.03⁎	0.002⁎⁎	0.8
Lesioned tissue f_M_	–	0.140 (0.023)	0.138 (0.035)	0.0009⁎⁎⁎	0.002⁎⁎	0.0007⁎⁎⁎	0.7
NASCT vs. lesioned tissue^a^	–	0.9	0.3	–	–	–	–

Longitudinal							
Change in NASCT f_M_	− 0.9% (7.0); p = 0.7	− 7.3% (5.2); p = 0.02⁎	+ 5.8% (10.2); p = 0.1	p = 0.004⁎⁎	p = 0.02⁎	p = 0.1	p = 0.002⁎⁎
Change in lesioned tissue f_M_	–	− 1.4% (7.6); p = 0.4	+ 3.1% (10.5); p = 0.2	p = 0.2	–	–	–

f_M_: myelin water fraction. NASCT: normal-appearing spinal cord tissue.

⁎ p ≤ 0.05.

⁎⁎ p ≤ 0.01.

⁎⁎⁎ p ≤ 0.001.

a Within-group paired Wilcoxon signed-rank test.

### 3.1 Cross-sectional

#### 3.1.1 Lesion identification

Four MS subjects had lesions spanning the whole cervical cord, and one had no detectable lesions. Two NMOSD subjects had lesions spanning the whole cord, and three had none. The number of lesional segments per subject did not differ significantly between groups (p = 0.1) (see Supplementary materials).

#### 3.1.2 f_M_ in NASCT and lesioned tissue (Fig. 2)

f_M_ was significantly reduced in the NASCT for both MS (on average − 9.66%, p = 0.03) and NMOSD (− 9.8%, p = 0.002) compared to controls. f_M_ in lesioned areas was also reduced in both MS (− 14.9%, p = 0.002) and NMOSD (− 16.4%, p = 0.0007). Neither NASCT nor lesioned tissue f_M_ differed between MS and NMOSD. There were no significant differences between NASCT and lesional areas within either patient group. Example maps are shown in Fig. 3.

**Fig. 2:**
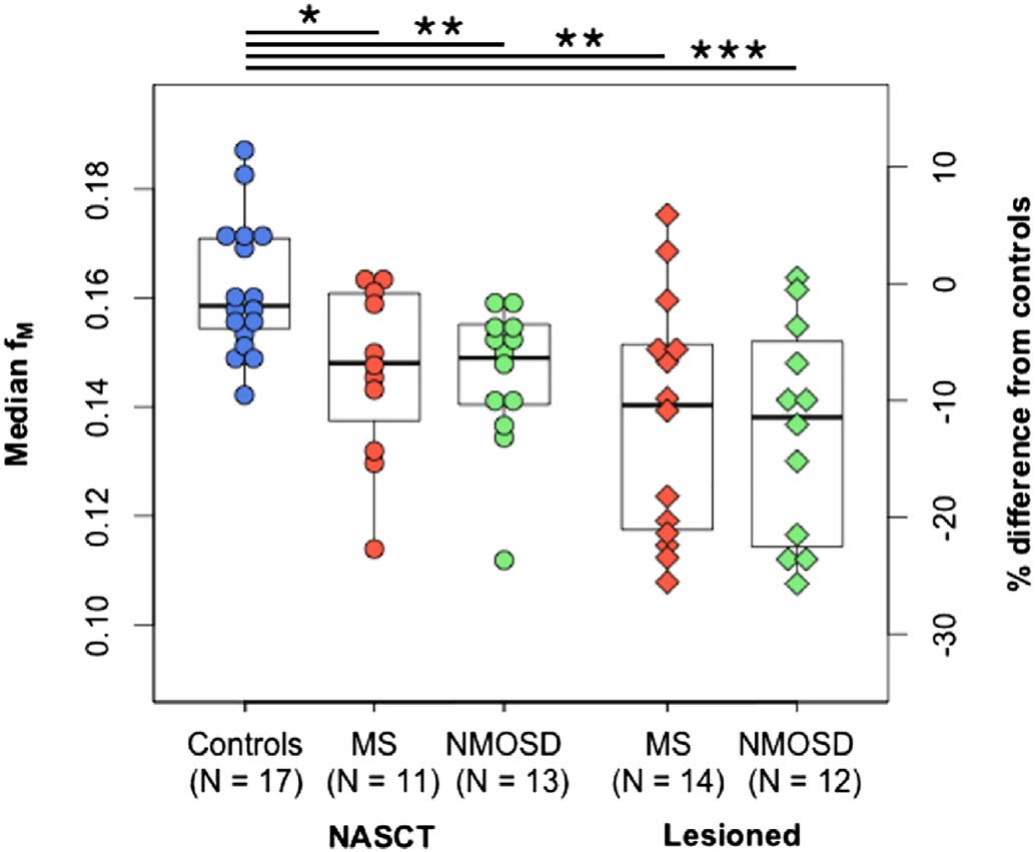
Median myelin water fraction (f_M_) for the cross-sectional sample, by group and tissue type: normal-appearing spinal cord tissue (NASCT) and lesioned segments. Right-hand axis shows percent difference of each data point from the control group average. Data points outside of boxplot whiskers are outliers, defined as being beyond 1.5 interquartile range of each quartile. *Significant at p ≤ 0.05, **p ≤ 0.01, ***p ≤ 0.001.

**Fig. 3:**
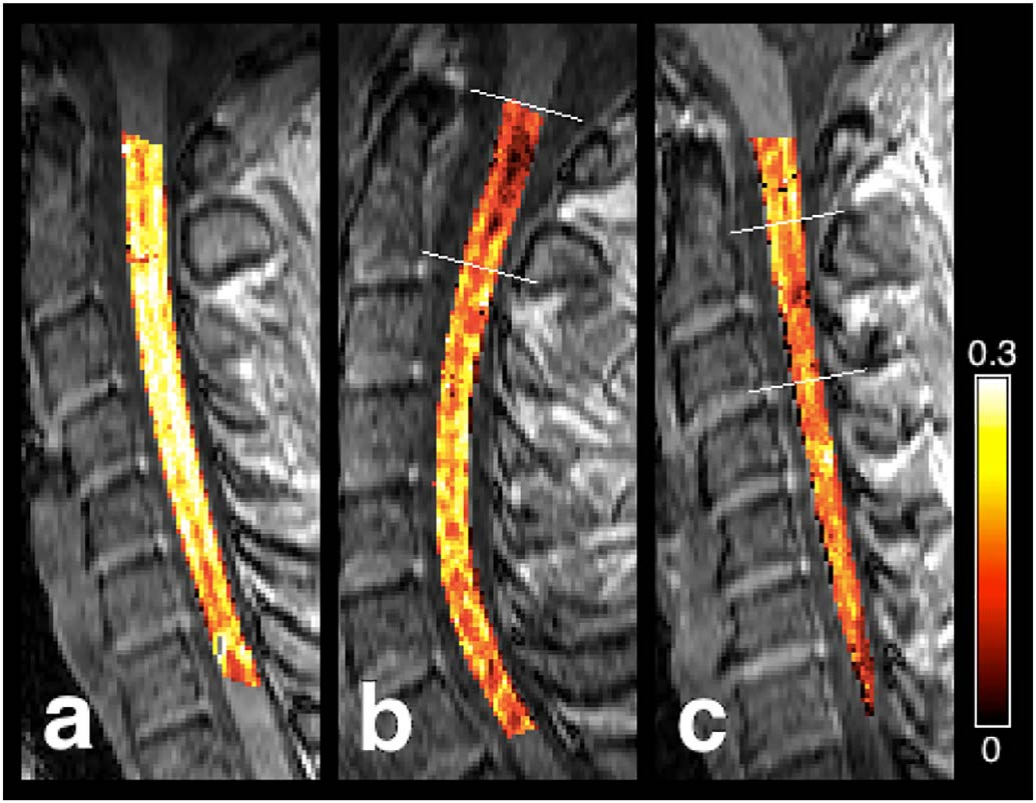
Example myelin water fraction (f_M_) maps overlaid on an SPGR scan. (a) Healthy control: female, 26 years old. (b) MS subject: male, 35 years old, EDSS 2, disease duration of 4 years, with lesions at the C1 and C2 levels. (c) NMOSD subject: female, 41 years old, EDSS 3.5, disease duration of 2 years, with a lesion at the C2/C3 level. White lines represent upper and lower limits for cord segments identified as lesioned. Reduced f_M_ is visible in lesions and along the length of the cord for both patients.

### 3.2 Longitudinal

Additional information regarding baseline metrics for the longitudinal subset can be found in Supplementary materials. The results observed at baseline overall held true for the subset of subjects who took part in the longitudinal part of the study. In the MS group, three subjects had an increase of 0.5 points on the EDSS, and two subjects a decrease of 0.5 points; all of them had a baseline EDSS ≤ 4.5. In the NMOSD group, one subject had an increase of 0.5 points from 5.5 to 6, and one had a one-point decrease from 7.5 to 6.5.

#### 3.2.1 Lesion identification

None of the subjects developed any new cervical cord lesions between baseline and follow-up. Within the longitudinal MS sample (n = 11), one subject had no lesions, therefore lesional analysis included 10 patients; 3 had entirely lesioned cords, therefore NASCT analysis only included 8 patients. Within the NMOSD group, all 8 subjects had some degree of NASCT tissue, and one had no lesions, therefore 7 patients were included in lesional analysis.

#### 3.2.2 Changes in f_M_ in NASCT and lesioned tissue (Fig. 4)

Median percent change in f_M_ was − 0.9% in controls. In NASCT, there was a greater reduction in the MS group (− 7.3%) compared to controls (− 0.9%, p = 0.02) and NMOSD (+ 5.8%, p = 0.002). There was no difference between controls and NMOSD. Within lesioned tissue, the MS group showed a median decrease of − 1.42%, and the NMOSD group increased by + 3.05%. A Kruskal-Wallis test revealed that there was no significant difference between change in NASCT in controls and change in lesioned tissue for both patient groups. Testing for significance of change within-group, only the decrease in NASCT in the MS group was significant (p = 0.02).

**Fig. 4:**
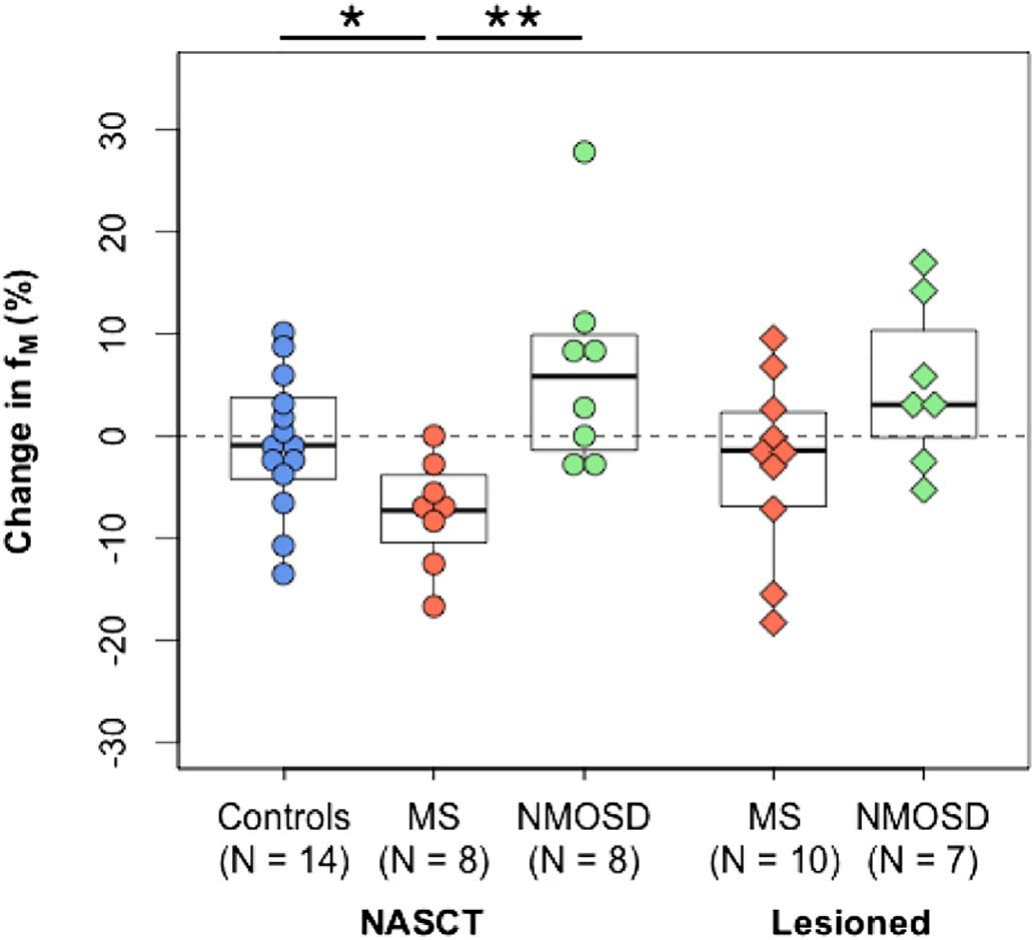
Percent changes in median myelin water fraction (f_M_) within normal-appearing spinal cord tissue (NASCT) and lesioned cord segments, over one year. Dotted line represents 0% change from baseline. Data points outside of boxplot whiskers are outliers, defined as being beyond 1.5 interquartile range of each quartile. *Significant at p ≤ 0.05, **p ≤ 0.01.

## 4 Discussion

Using myelin water imaging, we found that measures of myelin content are similarly reduced in MS and NMOSD compared to controls in cervical spinal cord lesions and in normal-appearing cervical spine tissue. At one-year follow-up, we observed a reduction in myelin content in NASCT in MS, but not in NMOSD, without intervening relapses. There was no significant change in myelin content in lesioned segments over time in either group. There were no within-group differences between NASCT and lesional areas for either MS or NMOSD.

Our findings complement those of a recent investigation by Matthews et al., which included the subjects enrolled in the present study (Matthews et al., 2015). Their results showed evidence of widespread non-lesional brain injury in the MS group only, using several MRI parameters including volumetric measures, myelin water imaging and diffusion tensor imaging. No changes in brain metrics were found after one year in NMOSD, while evidence of ongoing neurodegeneration was seen in the relapsing-remitting MS group.

The presence of diffuse abnormalities in the MS cervical cord is well-documented (Gass et al., 2015), although these may also be attributed to lesions not visible on conventional MRI (e.g. below the imaging resolution), or distal effects of damage to regions outside the cervical cord. Given comparable degrees of demyelination in the cord at baseline, the differences observed between MS and NMOSD groups over time may reflect distinct inflammatory and neurodegenerative processes (Kawachi and Lassmann, 2017), and may be consistent with the hypothesis of a primary neurodegenerative process in MS (Stys et al., 2012) that would be absent in NMOSD. Further evaluation of the effects of the location and extent of focal cord pathology are however required before claims can be made regarding the pathological substrates of these observations.

While the existence of diffuse damage in the brain in NMOSD is disputed, widespread abnormalities in the cervical spinal cord have been observed before (Jeantroux et al., 2012, Klawiter et al., 2012, Qian et al., 2011). Abnormal mean and perpendicular diffusivity parameters have been attributed to inflammation and demyelination, respectively, rather than axonal loss (Qian et al., 2011). Our findings bring further evidence for the presence of demyelination outside of lesional areas. Further, Klawiter et al. found abnormal diffusion parameters in tracts up- and downstream of lesioned areas, but not in those tracts that were unrelated to lesions (Klawiter et al., 2012). Secondary degeneration of white matter tracts following focal damage may therefore explain loss of myelin in adjacent areas. Since subjects in the present study were relapse-free for a minimum of 6 months before enrolment, it is likely that any lesion-related secondary changes would have already occurred at the time of baseline scan, hence the lack of chronic progression over the subsequent year. Alternatively, the abnormalities observed in NASCT may be the consequence of previous focal damage, resolved at the time of scanning but leaving residual abnormalities in the white matter not seen on conventional imaging, but to which myelin water imaging is sensitive. Our results of stable f_M_ in NASCT over one year suggest that, once these changes have occurred, normal-appearing areas are not subject to further chronic degeneration, which supports the observation that disease progression is absent clinically in NMOSD.

An important limitation of the current approach lies in the segmentation of lesioned areas, which include varying proportions of NASCT, and are therefore lesioned areas and not purely lesioned tissue per se. While this approach enables isolation of NASCT, it does not constitute lesion segmentation per se, and thus limits the specificity of this measure to lesional pathology. This may contribute to explain the lack of significant baseline differences between NASCT and lesioned areas in each patient group, as would be expected, although the observed extent of damage to NASCT areas is another contributing factor.

We did not differentiate between upstream and downstream lesional influences on normal-appearing tissue, and results obtained in lesional areas are likely to reflect heterogeneity in the size, type, and age of lesions. Moreover, there is heterogeneity of normal myelin concentrations at different levels of the cervical cord due to variations in white to grey matter ratio (Fradet et al., 2014, Kolind and Deoni, 2011). However, the distribution of lesions across cord levels was uniform within and between each disease group, thus mitigating possible bias in comparing unmatched regions that are expected to vary in myelin content.

Half of the MS and all of the NMOSD subjects were on immunomodulatory and immunosuppressant treatments, respectively. While all included patients were in remission for the course of the study, disease-modifying therapies are likely to mediate background inflammatory activity, and thus may affect changes in myelin content over one year compared to patients not currently on treatment, although discussions on the effects of DMTs on spinal cord pathology in the current context remain speculative. Characterisation of the influence of volume changes (i.e. atrophy) on the f_M_ measurements would also be of interest to allow description of the relationship between possible volume loss and changes in myelin content.

Limitations to this study also include the very small sample size, especially for the longitudinal sample, and resulting heterogeneity in age and clinical profiles within the patient groups. Therefore, results should be regarded as preliminary and warrant replication in larger samples. However, detection of significant changes over a short period in a small population strongly supports the potential use of cervical cord myelin water imaging as a marker of ongoing demyelination in MS.

## 5 Conclusion

Using a myelin water imaging protocol, we detected cross-sectional differences in normal-appearing tissue in NMOSD and relapsing-remitting subjects compared to healthy controls. There was a significant decrease in f_M_ over one year in the MS NASCT, suggesting that chronic ongoing demyelination in non-lesional areas may occur in relapsing-remitting subjects. These results also attest to the ability of mcDESPOT to show evidence of degenerative processes in the relapsing-remitting MS cord over a relatively short follow-up duration. Further longitudinal investigations should address the link between such changes and progression of disability in order to establish its potential use as a clinically relevant marker of pathology. In contrast, despite severe diffuse damage at baseline, no significant change in myelin content was seen in NMOSD in normal-appearing areas over time. This observation lends new evidence to the theory that subclinical disease activity is absent in NMOSD.

## Appendix A Supplementary data

Supplementary materials include: Table S1. Population characteristics and baseline MRI metrics for the longitudinal sample; Figure S1. Distribution of lesions by group. Image 1
