# Supplementary material for: Cervical cord myelin water imaging shows degenerative changes over one year in multiple sclerosis but not neuromyelitis optica spectrum disorder
Source: Neuroimage Clin. 2017 Jun 16;16:17–22. doi: 10.1016/j.nicl.2017.06.019 (PMC5503831; doi:10.1016/j.nicl.2017.06.019)
Supplement: Supplementary file 1 — Supplementary materials include: Table S1. Population characteristics and baseline MRI metrics for the longitudinal sample; Figure S1. Distribution of lesions by group. [file mmc1.docx]

**­Supplementary materials**

|  | Controls | MS | NMOSD | Kruskal-Wallis | MS vs. controls | NMOSD vs. controls | NMOSD vs. MS |
| --- | --- | --- | --- | --- | --- | --- | --- |
| N (Sex) | 14 (3M) | 11 (3M) | 8 (0M) | - | - | - | - |
| Age (years) | 51 (26-76) | 41 (28-62) | 46 (27-76) | - | - | - | .6 |
| Disease duration (months) | - | 54 (26-156) | 65 (24-186) | - | - | - | .9 |
| Baseline EDSS | - | 2 (1-5) | 4.75 (2-7.5) | - | - | - | .04* |
| 1-year EDSS | - | 2.5 (1-5) | 5 (2-6.5) | - | - | - | .06 |
| Lesioned segments per subject | - | 4 (0-7) | 3 (0-5) | - | - | - | .2 |
| Number of subjects with NASCT |  | 8 | 8 | - | - | - | - |
| Number of subjects with ≥ 1 lesion |  | 10 | 7 | - | - | - | - |
| Number of subjects with no lesions |  | 1 | 1 | - | - | - | - |
| Time between scans (months) | 12 (10-14) | 12 (11-14) | 12 (11-15) | .3 | - | - | - |
| NASCT f_M_ | 0.159 (0.016) | 0.148 (0.018) | 0.150 (0.008) | .02* | .04* | .01* | .3 |
| Lesioned tissue f_M_ | - | 0.143 (0.033) | 0.149 (0.018) | .01* | .006** | .046* | .6 |
| NASCT vs. lesioned tissue^†^ | - | 1 | .7 | - | - | - | - |

Table S1. Population characteristics (median (range)) and baseline MRI metrics (median (interquartile range)) for the longitudinal sample. Group comparisons are performed with the Kruskal-Wallis test, and Dunn’s test for post-hoc comparisons. Where only pairwise MS vs. NMOSD comparisons are appropriate, results from the Mann-Whitney U test are reported in the last column. ^†^Within-group paired Wilcoxon signed-rank tests. *Significant at p ≤ .05, *p ≤ .01. f_M_: myelin water fraction. NASCT: normal-appearing spinal cord tissue.


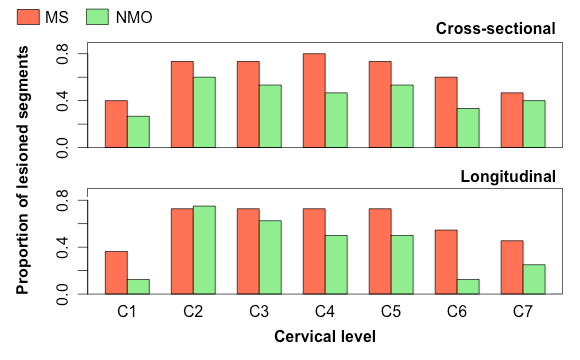
Fig. S1. Distribution of lesion location for each group, for the cross-sectional and longitudinal samples. At baseline, in the MS group, a total of 67/105 segments (65%) were identified as containing lesioned tissue. Four MS subjects had lesions spanning the whole cervical cord, and one had no detectable lesions. In the NMOSD group, a total of 47/105 segments (45%) contained lesions. Two NMOSD subjects had lesions spanning the whole cord, and two had none. The number of lesional segments did not differ significantly between groups (p = .1). In the follow-up subset, a total of 47/77 segments (61%) contained lesions for the MS group. Three subjects had lesions covering the whole cervical cord, and one had none. In the NMOSD group, 23/56 segments (41%) were counted as lesioned; one subject had no lesions. The number of lesioned segments did not differ between groups (p = .2).
